# Influence of a metaphyseal sleeve on the stress-strain state of a bone-tumor implant system in the distal femur: an experimental and finite element analysis

**DOI:** 10.1186/s13018-020-02025-6

**Published:** 2020-12-09

**Authors:** Jian-jun Li, Dong-mu Tian, Li Yang, Jing-yu Zhang, Yong-cheng Hu

**Affiliations:** 1 Tianjin Medical University, 22 Qixiangtai Road, Tianjin, People’s Republic of China; 2 Department of Bone Oncology, Tianjin Hospital, 406 Jiefang Southern Road, Tianjin, People’s Republic of China; 3 Department of Bone Trauma, Second Hospital of Tangshan, 22 Jianshe North Road, Tangshan, Hebei People’s Republic of China; 4 Beijing Weigao Yahua Artificial Joint Development Company, 7 Niuhui Street, Shunyi, Beijing, People’s Republic of China; 5 Department of Bone Oncology, Second Hospital of Tangshan, 22 Jianshe North Road, Tangshan, Hebei People’s Republic of China

**Keywords:** Distal femur, Sleeve, Finite-element analysis, Strain gauge, Stress shielding

## Abstract

**Background:**

Aseptic loosening of distal femoral tumor implants significantly correlates with the resection length. We designed a new “sleeve” that is specially engaged in the metaphysis at least 5 cm proximal to the knee joint line to preserve as much bone stock as possible. This study investigates the influence of a metaphyseal sleeve on the stress-strain state of a bone tumor implant system in the distal femur.

**Methods:**

Cortex strains in intact and implanted femurs were predicted with finite element (FE) models. Moreover strains were experimentally measured in a cadaveric femur with and without a sleeve and stem under an axial compressive load of 1000 N. The FE models, which were validated by linear regression, were used to investigate the maximal von Mises stress and the implanted-to-intact (ITI) ratios of strain in the femur with single-legged stance loading under immediate postoperative and osseointegration conditions.

**Results:**

Good agreement was noted between the experimental measurements and numerical predictions of the femoral strains (coefficient of determination (*R*^2^) ≥ 0.95; root-mean-square error (RMSE%) ≈ 10%). The ITI ratios for the metaphysis were between 13 and 28% and between 10 and 21% under the immediate postoperative and osseointegration conditions, respectively, while the ITI ratios for the posterior and lateral cortices around the tip of the stem were 110% and 119% under the immediate-postoperative condition, respectively, and 114% and 101% under the osseointegration condition, respectively. The maximal von Mises stresses for the implanted femur were 113.8 MPa and 43.41 MPa under the immediate postoperative and osseointegration conditions, which were 284% and 47% higher than those in the intact femur (29.6 MPa), respectively.

**Conclusions:**

This study reveals that a metaphyseal sleeve may cause stress shielding relative to the intact femur, especially in the distal metaphysis. Stress concentrations might mainly occur in the posterior cortex around the tip of the stem. However, stress concentrations may not be accompanied by periprosthetic fracture under the single-legged stance condition.

## Introduction

The distal femur is a common site for primary bone tumors [1]. Endoprosthetic replacement of the distal femur is the most commonly used reconstruction method after tumor resection [2]. The advantages of reconstruction with implants include a relatively durable and stable reconstruction, maintenance of joint range of motion, and patient activity resumption in a timely fashion [3]. However, several complications have been reported, particularly resulting from stem loosening [4].

Aseptic loosening, which is the main reason for failure of distal femoral replacements using current modular megaprostheses [5], significantly correlates with the resection length and total prosthesis length/stem length ratio, as described in some studies [6, 7]. Three factors may contribute to aseptic loosening of distal femoral implants in relation to bone defect situations. First, the offset distance is greater in the proximal femur than in the distal femur; an increase in offset will increase the bending moment around the stem [8]. Therefore, the bending moment will be greater in the proximal femur than in the distal femur, which may explain why the probability of aseptic loosening increases as the percentage of the distal femur replaced increase [6, 8]. Second, an increase in bone resection length might lead to a reduction in the area of main fixation and a reduced contact area between the implant and bone, which may be harmful to the long-term stability of distal femoral implants [5]. In addition, aseptic loosening has been linked to bone loss secondary to stress shielding [9], which can lead to excessive stresses in the bone cement or at the interfaces between the bone and the prosthesis stem [10]. Conlisk et al. [11] indicated that a longer stem, which is indirectly correlated with bone resection length [6], may result in more stress shielding than a shorter stem.

The metaphyseal sleeve was introduced with a low rate of loosening, providing a direct cementless fixation option at the metaphysis [12] and thus, complying with the principles of conservation to preserve as much bone stock as possible [13]. Lin et al. [14] reported that in patients with giant cell tumor of bone (GCTB) in the distal femur, 80% of the maximal longitudinal diameter measurements of tumors in the distal femur were 4.4–8.9 cm and 80% of the shortest distance from the articular surface values were within 0.01–0.75 cm. So, excessive bone would be removed for the reconstruction with the current tumor prosthesis. Wang et al. [15] measured the morphological parameters of the supracondylar femur to classify supracondylar femurs and to provide theoretical guidance for the development of distal femoral prostheses. On the basis of a study of the metaphysical anatomical structure of distal femurs, we designed a new “sleeve” that is specially engaged in the metaphysis at least 5 cm proximal to the knee joint line. The sleeve, which is engaged in a particular area of the metaphysis, has not been widely adopted or promoted by others for distal femur reconstruction after resection of tumors located near the knee line, especially in Chinese patients, and perhaps in Asian patients in general. In addition, the effect of the sleeve on the strain distribution in the distal femur is poorly understood.

Stress analysis may help to reduce the surgical use of mechanically unsound implant designs and may also help to improve the design of existing prostheses [16]. The current study was designed to investigate the influence of a metaphyseal sleeve on the stress-strain state of a bone-implant system in the distal femur. The hypothesis was that the stress distribution in the bone around the femoral component may be close to that in the intact femur.

## Materials and methods

### Experiment with strain-gauge measurements

#### Specimen preparation from a cadaveric femur

One intact, fresh, right cadaveric femur from a 75-year-old women (weight 70 kg, height 170 cm) who died of nonorthopedic diseases was selected for the biomechanical tests, in where the surface strains of intact and implanted femur were measured [17]. The details of specimen preparation have been described in Kim et al. [18]. The specimen had a length (defined as the distance between the most distal point on the lesser trochanter and the center of the knee joint) of 421.5 mm.

#### Meatal sleeve and stem

**Fig. 1:**
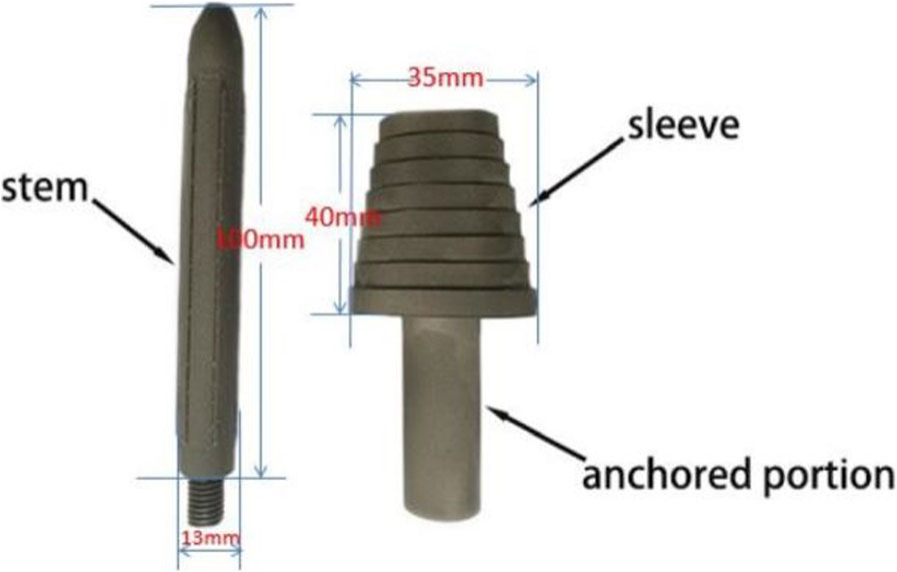
Sleeve and stem

The sleeve is conical and stepped. The outer surface of the oval-shaped terraces is grit-blasted to induce biological ingrowth. The sleeve distal width (major diameter) is 35-mm medial to lateral. The sleeve height is 40 mm. The stem with a grit-blasted coating is straight, tapered, and fluted. The diameter and length of the stem are 13 mm and 100 mm, respectively. A straight fluted stem provides the best initial resistance to rotational stresses [19]. The sleeve and stem are manufactured from titanium alloy (Ti-6Al-4 V) (Beijing Weigao Yahua Artificial Joint Development Company, Beijing, People’s Republic of China) and are shown in Fig. 1. (The anchor was designed to be covered by cement to fix the specimen in the experiment.)

#### Strain-gauge attachment

**Fig. 2:**
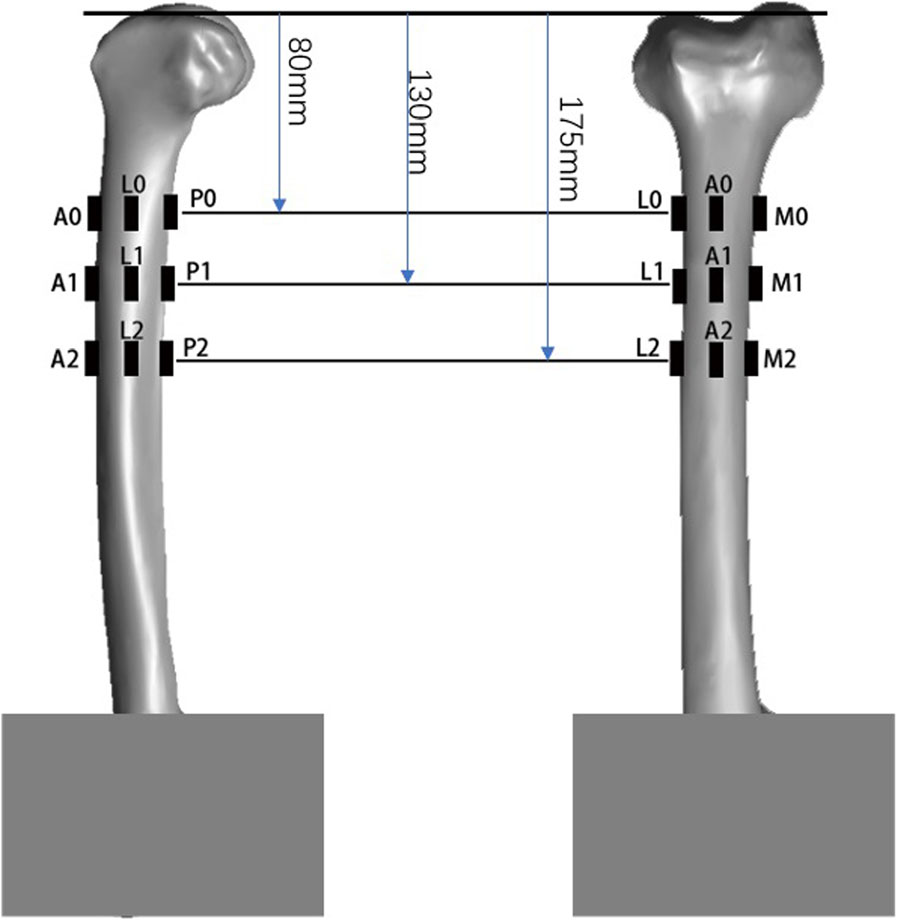
Distal femur with locations of strain gauges. Bone strains were measured with 12 gauges glued at the anterior (A0, A1, and A2) and posterior (P0, P1, and P2) sides and medial (M0, M1, and M2) and lateral (L0, L1, and L2) sides of the femur

A system of reference axes was marked following the same approach reported in Ruff and Hayes [20], which allowed reproducible positioning of the strain gauges and alignment during testing. Strain gauges were glued at three different levels on the lateral (L), medial (M), anterior (A), and posterior (P) aspects of the femur (Fig. 2). The locations of the measuring points for which the most distal level of the lateral condyle served as a length reference were as follows: #0, 80 mm; #1, 130 mm; #2, 175 mm proximal to the reference. Theoretically, the strain gauges should be attached to the same area in the femur with and without a sleeve and stem.

Eight uniaxial strain gauges (BX120-3AA; Yiyang Strain and Vibration Testing Technology Co., Ltd., Beijing, China) and four biaxial gauges (BX120-3BA; Yiyang Strain and Vibration Testing Technology Co., Ltd., Beijing, China) were attached to the surface of the diaphyseal and epiphyseal regions of the specimen with 502 superglue (7147; Deli Group Co., Ltd., Ningbo, China) [21], after the attachment site was cleaned and degreased in accordance with a standard protocol [22].

All strain gauges were connected to a data acquisition system (DH5922D, Donghua Testing Technology Co., Ltd., Jiangsu, China), which was connected to a PC to record the data with the DHDAS software (Donghua Testing Technology Co., Ltd., Jiangsu, China).

#### Axial compression testing

The distal end of the femur (1.5 cm height) was anchored using polymethylmethacrylate cement (Medantal, New Century Dental Materials Co., Ltd., Shanghai, China), and the proximal end of the femur was mounted in a multiaxial clamping system, which enabled rigid fixation during testing. The femur with a medullary canal diameter 13 mm was osteotomized in the metaphysis, where 5 cm of the distal femur was removed. Straight cylindrical reamers were then used in 0.5 mm increments up to 12.5 mm. The sleeve and stem were then implanted into the specimen.

**Fig. 3:**
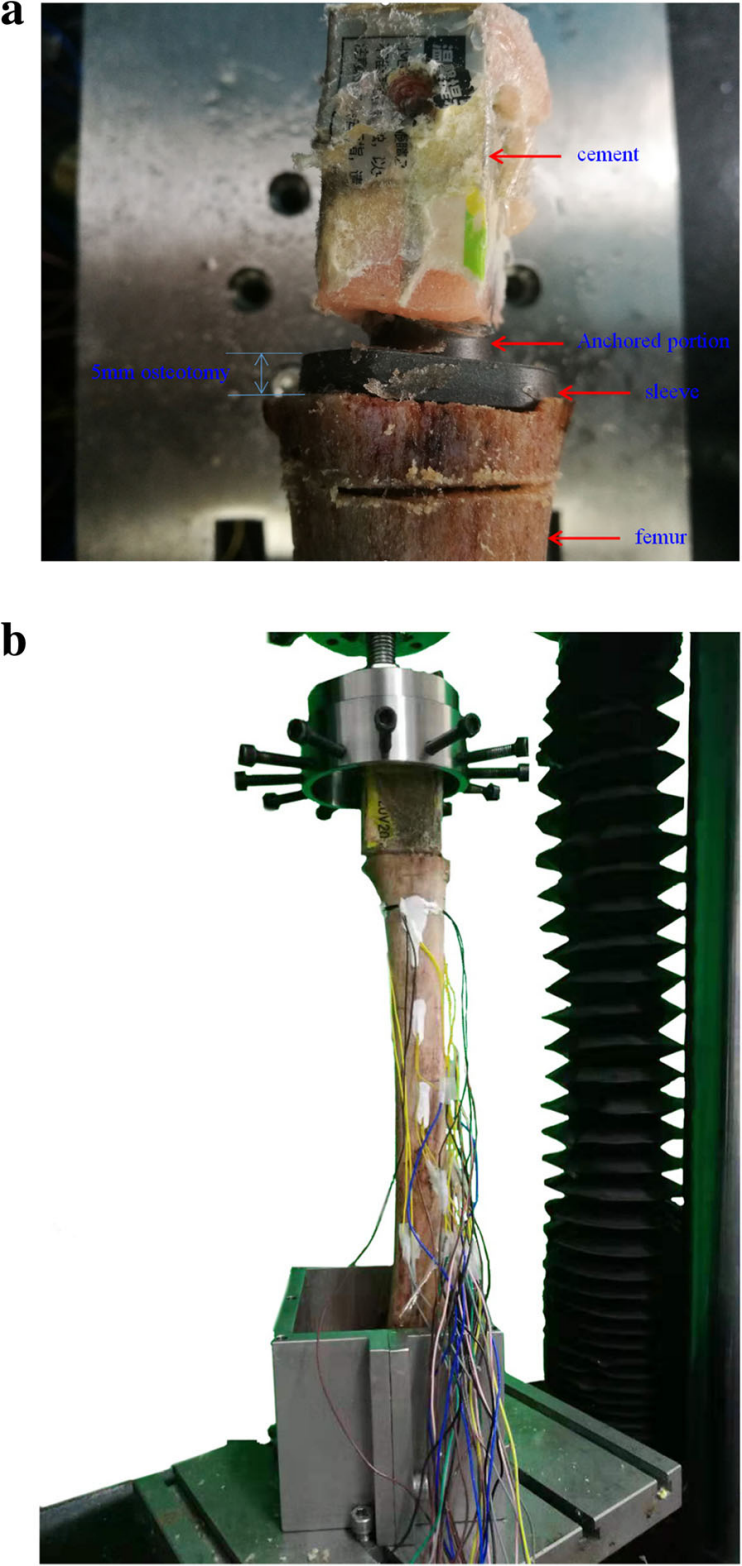
Experimental model of instrumented implanted femur: (**a**) sleeve with anchored portion implanted into femur in preliminary experiment, (**b**) mechanical testing mode

Using a test machine (ElectroForce 3100, Bose, Framingham, Mass, America), the sleeve and stem were pressed into the femurs by applying a 1700 N load to imitate the press fit achieved in vivo [23]. The 40-mm straight cylindrical block connected with the trapezoid component was anchored with cement (Fig. 3a). The instrumented implanted femur is shown in Fig. 3b.

For the specimen, a vertical load up to a maximum of 1000 N was applied to the distal cement without damaging the bone [21]. Strains were measured following the protocol described in a previous study [24]. Strains on the specimen must be measured five times to ensure reproducibility. The cadaveric bone specimen was kept moist using saline solution during testing.

### FE analyses

#### Three-dimensional geometry of the femoral bone

The femur was scanned via computed tomography (CT; Optima CT660, General Electric Co., USA). Details of the CT axial scanning protocol are provided in Table 1. The CT datasets were segmented (Mimics 14® and 3-Matic®, Materialise, Leuven, Belgium), nonuniform rational B-spline (NURBS) models were subsequently developed (Geomagic Studio 2013 , Geomagic, Inc., Research Triangle Park, NC, USA), a 3D model of cortical and cancellous bone was obtained (SolidWorks 2016, Dassault Systemes SolidWorks Corp, Waltham, USA), and twelve regions of interest were identified in the intact femur to simulate the locations where the strain gauges were attached in the experiments. Each region was the shadow of a rectangular area with a width of 2.3 mm and length of 3 mm determined cutoff rule-projection in SolidWorks.

**Table 1:** The CT protocol details

Scanning mode	Helical
Slice thickness	3.75 mm
Reconstruction spacing	3.75 mm
Pixel dimension	0.49 mm
Tube current	200 mA
Voltage	100kVP

#### Three-dimensional model of the metal sleeve and stem

The sleeve and stem geometries were developed using SolidWorks and designed to imitate the commercial knee implant system (Weigao China) used in the experimental portion of this study. The models of the femoral components were created with the exact same scale and assembly used in the experimental setup, including the sleeve and stem. The implant components and the bone geometry were exported to Abaqus CAE 2018 (Simulia, Providence, RI, USA) for FE analysis.

#### Material properties, boundary conditions, interface conditions

A Young’s modulus of 4.5 GPa was assigned to the cortical bone, which was referenced from previous study [25], to mimic poorer quality human bone because of the age of the cadaveric femur used in this study. An implant Young’s modulus of 110 GPa (titanium alloy) was assigned to the implant, following the approach by El-Zayat et al. [26]. A Poisson’s ratio of 0.3 was assigned to all materials [26], which were assumed to behave in a linear elastic, isotropic, and homogeneous manner. The Young’s moduli and Poisson’s ratios applied to all structures in this study are presented in Table 2.

**Table 2:** Type and number of elements and Young’s modulus (E) and Poisson’s ratio for FE models

Solid model	Number of elements	Elements	E (GPa)	Poisson’s ratio
Cortical bone	63,296	C3D10M	4.5(16.7)	0.3
Cancellous bone	57,240	C3D10M	0.104	0.3
Cortical bone with 5 cm resection	70,317	C3D10M	4.5(16.7)	0.3
Sleeve	10,375	C3D10M	110	0.3
Stem	6,311	C3D10M	110	0.3

Data in parentheses are Young’s modulus applied to the FE models after validation

For the boundary conditions, the proximal femoral cement block was modeled as a rigid, fixed support, and all its translations/rotations were fixed. For the FE model of the intact femur, the interface between the cortical and cancellous bone was simply assumed to have bonded contact; however, the contact mode for the implanted femur would be referred to as a frictional case. The sliding formulation was small sliding. A coefficient of friction μ = 0.3 [27] was used for the interfaces between the sleeve, stem, and cortical bone. The Coulomb friction model was used.

The axial force in the bone-tumor implant was applied through the top-facing surface of the cement block, replicating the experimental study where the actuator presses down on the femur; the location on the condyles of the intact femur where the axial force was applied was near the femoral intramedullary guiding rod entry point [28].

After validation of the FE models, a load of 2030 N [29, 30] was applied to simulate loading conditions under a single-legged stance [31]. The femur prosthesis interface was considered frictional and bonded to simulate the immediate postoperative period and osseointegration, respectively [32]. A Young’s modulus of 16.7 GPa was assigned to the cortical bone to construct a healthy femur with good bone quality [11].

#### Mesh and convergence

Automatic meshing of the models was performed, and the meshes were built from 10-node modified quadratic tetrahedron elements (C3D10M). An element size of 3 mm was applied to all bones and stems, whereas an element size of 3.1 mm was applied to the sleeve. A convergence study of the maximum displacements in FE models showed that a further reduction in element edge length would produce a negligible (< 1%) change, while dramatically increasing the simulation runtime. The element type and number of elements in the FE models are described in Table 2.

### Indicators

Strains at each measurement point were investigated in the medial, lateral, anterior, and posterior aspects of the diaphyseal and metaphyseal region of the femur. The maximal principal strains in the FE models corresponding to the experimental strain measurement sites were obtained.

#### ITI ratios of the femoral strain

The percentage change in strain was calculated for the implanted femur (including immediate postoperative and osseointegration conditions) from the reference value before implantation (intact femur) in the same location [33]. The result was defined as the implanted-to-intact ratio (ITI ratios):

ITI=Immediate postoperation strain or osseointegration/Intact strain

The ITI ratios were computed for each strain measurement location. An ITI ratio of 100% indicates that no alteration with respect to the physiological condition. An ITI ratio lower than 100% indicates stress shielding. An ITI ratio larger than 100% indicates an increased strain state or possible stress concentration.

#### Maximal von Mises stress

von Mises stress is a convenient positive scalar value of stress, which is ideal for comparison of the overall stress magnitude [9]. Maximal von Mises stress is the peak value of stress for the overall model of components, which may be used to quickly determine the most dangerous areas in the model. Periprosthetic or prosthetic fracture is considered when the maximal von Mises stress is beyond the femoral strength or yield strength of titanium alloy.

### Statistical analysis

The mean and standard deviation of strains under axial compression were examined according to the FE and experimental groups. Simple linear regression analysis was used to evaluate the correlation between the maximal principal strains predicted by the FE models and the measured strains. The coefficient of determination (*R*^2^) is an important parameter for determining the degree of linear-correlations between variables (goodness of fit) in regression analysis. A higher *R*^2^ value with good determinant power (0.72–0.8) suggests a better fit [34]. The root-mean-square error (RMSE) is an additional indicator of the overall absolute difference between numerical and experimental strains, which was calculated and expressed as a percentage (RMSE %) of the peak values of the measured principal strains. *P* < .05 was used as the criterion for statistical significance. All statistical analyses were performed using the SPSS software (version 23.0; SPSS, Chicago, IL, USA).

## Results

### Verification of the FE models of the intact and implanted femurs

A total of 24 strains were used to assess the validity of the FE simulations of the intact femur, and 24 strains were used for the femur implanted with the sleeve and stem. Negative values represent compressive strains on the concave side, whereas positive values represent tensile strains on the convex side at all levels. Descriptive statistics were used, and the results are shown in Table 3. For the intact femur, the experimental tensile strain (average = 200.92 microstrain) was 1.5 times the simulated tensile strain (average = 134.07 microstrain). The experimental compressive strain (average = − 519.40 microstrain) was 2.5 times the simulated compressive strain (average = − 211.18 microstrain). For the implanted femur, the mean numerical tensile strain was 82.76 microstrain, and the mean experimental tensile strain was 96.52 microstrain, corresponding to an increase of 17%. The experimental compressive strain (average = − 204.84 microstrain) was 1.7 times the numerical compressive strain (average = − 120.43 microstrain).

**Table 3:** Principal strains in the intact femurs of FE and experiment

	Intact femur		Implanted femur	
	FE	Experiment	FE	Experiment
Tensile strains	134.07 ± 71.79	200.92 ± 56.56	82.76 ± 26.96	96.52 ± 25.32
Compressive strains	− 211.18 ± 30.06	− 519.40 ± 104.89	− 120.43 ± 93.89	− 204.84 ± 125.86

The average strain was computed for each strain measurement location. The values are given as the mean and the standard deviation

**Fig. 4:**
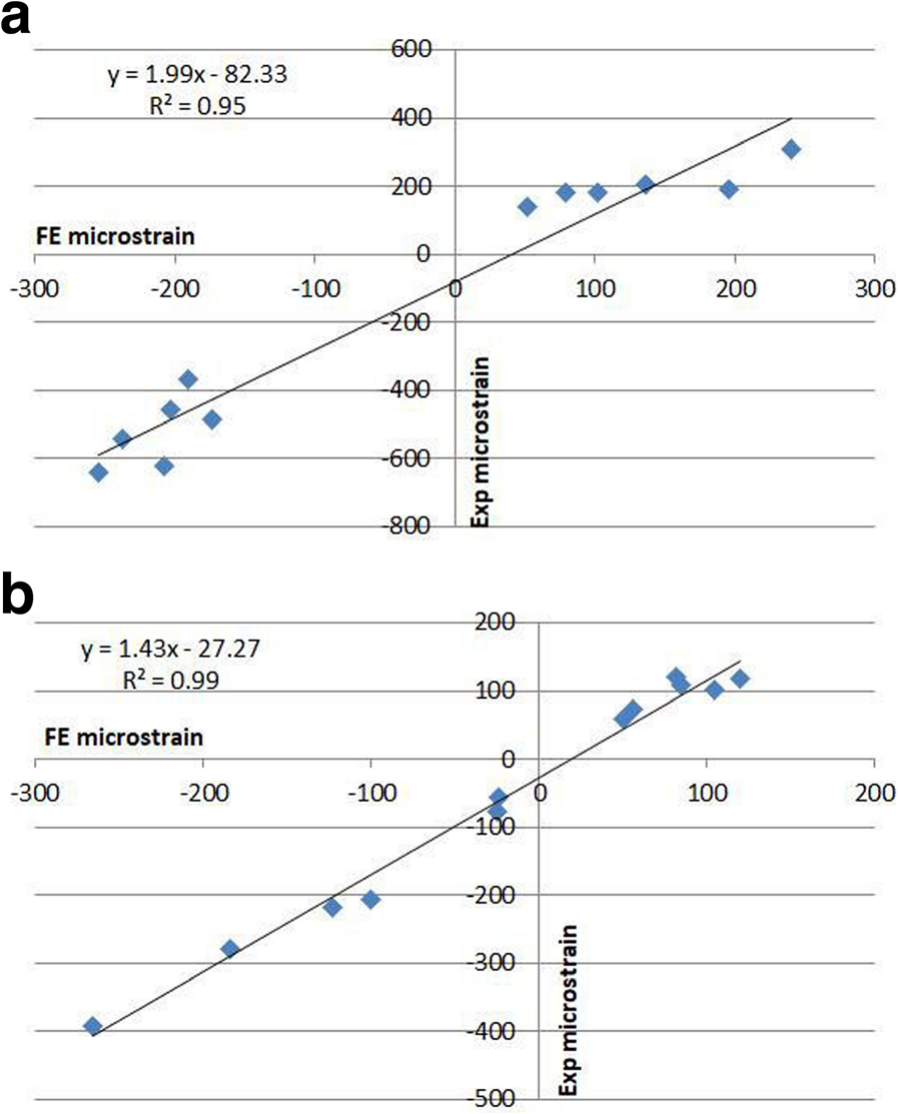
Linear regression analyses were performed for FE and mean measured strains. The graphs show the linear regression results for the strains in **a** intact femur, **b** implanted femur

Linear regressions were performed to determine the overall correspondence between the mean values of the experimental (*S*_EXP_) and numerical principal strains (*S*_FE_). *S*_EXP_ and *S*_FE_ were treated as dependent variables and independent variables, respectively. The results are shown in Table 4 and Fig. 4. A correlation for the intact femur was obtained with the following regression parameters: *S*_EXP_ = 1.99 × S_FE_ − 82.33, *R*^2^ = 0.95 (*P* < 0.05). A correlation for the implanted femur was obtained with the following regression parameters: *S*_EXP_ = 1.43 × S_FE_ − 27.27, *R*^2^ = 0.99 (*P* < 0.05). Excellent correspondence between the measured and FE strains was obtained for the two analyzed models because both *R*^2^ values were greater than 0.8, which is quite close to 1.

**Table 4:** The validation parameter of linear regression analysis, comparing measured strains and FE strains

	Intact femur	Implanted femur
R^2^	0.95	0.99
RMSE	91.72	22.43
RMSE%	14%	6%

RMSE% are calculated as a percentage of the maximum measured value

Very low error values (RMSE < 10% of the highest measured strain) were found for the implanted femur. However, the errors were larger for the intact femur (RMSE > 10% of the highest measured strain).

### Strain distribution in the intact femur

Representative strain data for the intact femur when subjected to a vertical force of 2030 N with the femoral shaft at 12° of adduction are shown in Table 5. The principal strains along the medial side of the intact femoral cortex under a simulated single-legged stance were compressive and ranged from 110.4 to − 128.1 microstrain (average = 119.13 microstrain). Principal tensile strains along the lateral cortex ranged from 52.55 to 105.8 microstrain (average = 76.46 microstrain). Principal compressive strains along the posterior cortex ranged from 96.1 to 138.3 microstrain (average = 121.43 microstrain). Principal tensile strains along the anterior cortex ranged from 32.62 to 160.7 microstrain (average = 81.25 microstrain). The strain decreased from distal to proximal in the anterior, lateral, and medial sides of the intact femurs under load. In contrast, the strain increased from distal to proximal on the posterior side. The highest value was 160.7 microstrain at A0, whereas the smallest value was 32.62 microstrain at A2.

**Table 5:** Strains of femur model with and without implant (μɛ) in single-legged stance

Side of bone	Location	Intact femur	Implanted femur			
			Immediate-postoperation (ITI)		Osseointegration (ITI)	
Anterior	A0	160.7	34.82 (0.22)		26.54(0.17)	
	A1	50.42	27.61 (0.55)		17.46(0.35)	
	A2	32.62	16.46 (0.50)		13.07(0.40)	
Posterior	P0	− 96.1	− 21.72(0.23)		− 12.65(0.13)	
	P1	− 129.9	− 85.11(0.66)		− 43.05(0.33)	
	P2	− 138.3	− 152.3(1.10)		− 157.2(1.14)	
Lateral	L0	105.8	29.24(0.28)		22.36(0.21)	
	L1	71.03	52.67(0.74)		31.3(0.44)	
	L2	52.55	62.62(1.19)		52.93(1.01)	
Medial	M0	− 128.1	− 16.9(0.13)		− 13.27(0.10)	
	M1	− 118.9	− 63.1(0.53)		− 31.53(0.27)	
	M2	− 110.4	− 108(0.98)		− 96.46(0.87)	

*ITI* the implanted-to-intact ratios computed for each strain measurement location in the femur

### Strain distribution in the implanted femur

**Fig. 5:**
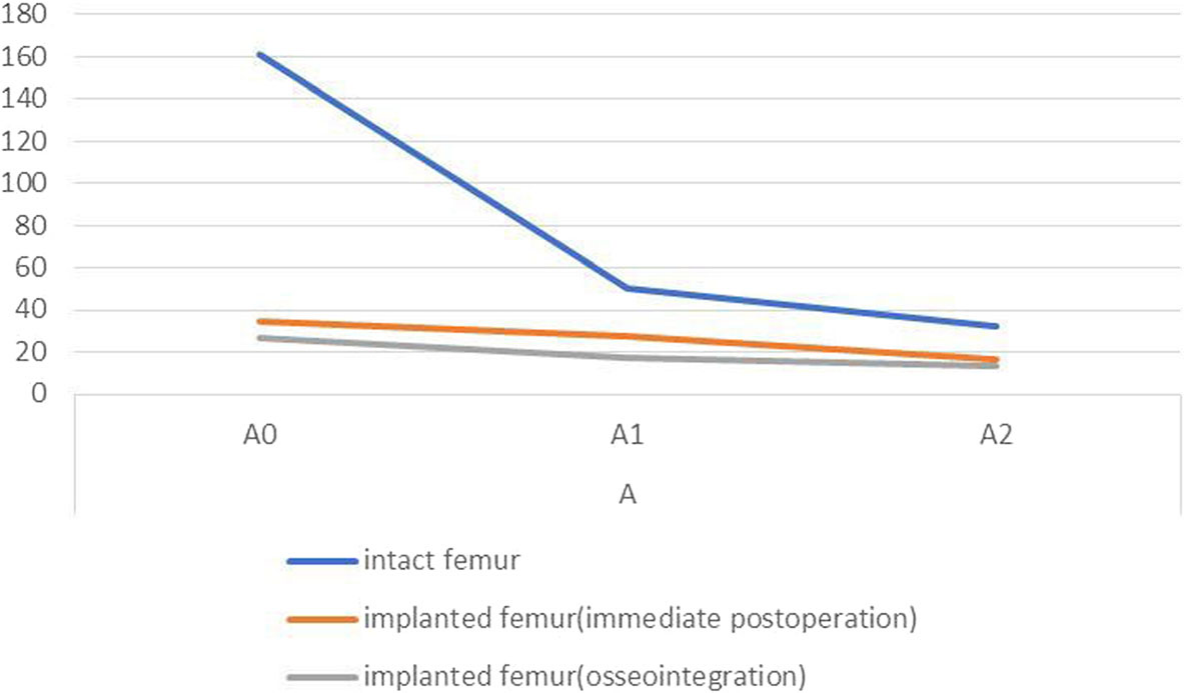
FE values of strain of intact and implanted femur at anterior region

**Fig. 6:**
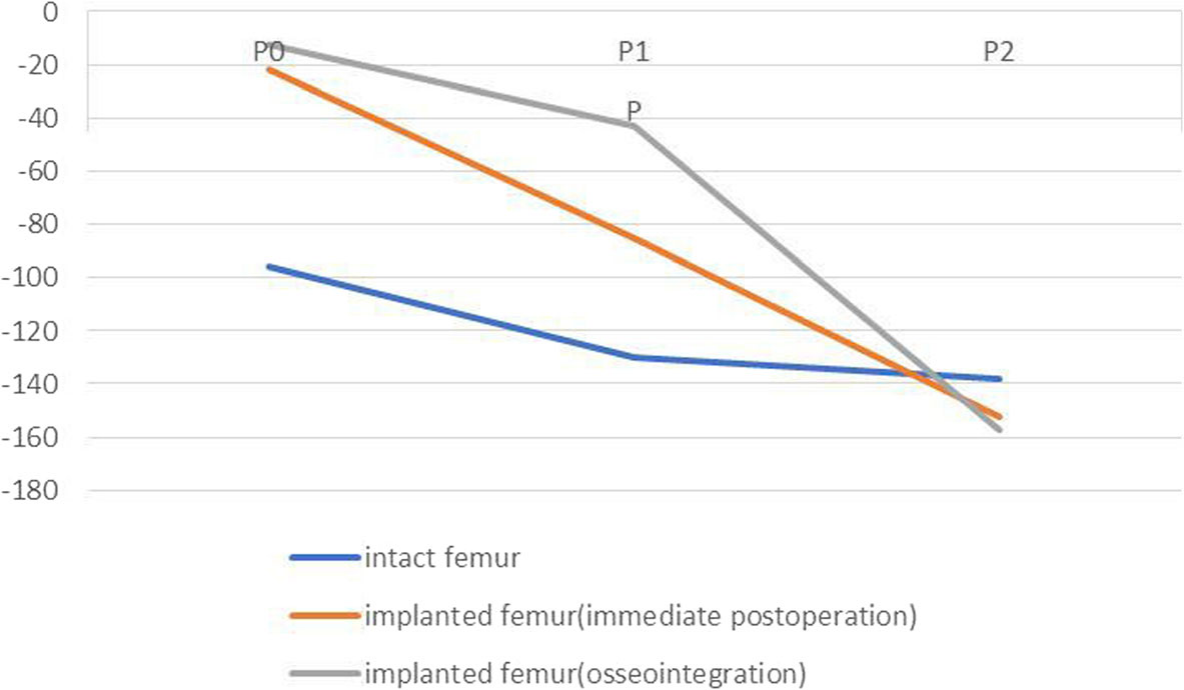
FE values of strain of intact and implanted femur at posterior region

**Fig. 7:**
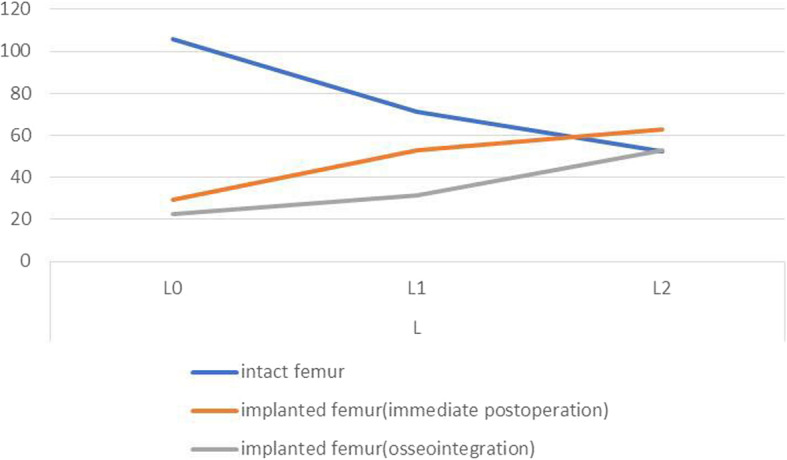
FE values of strain of intact and implanted femur at lateral region

**Fig. 8:**
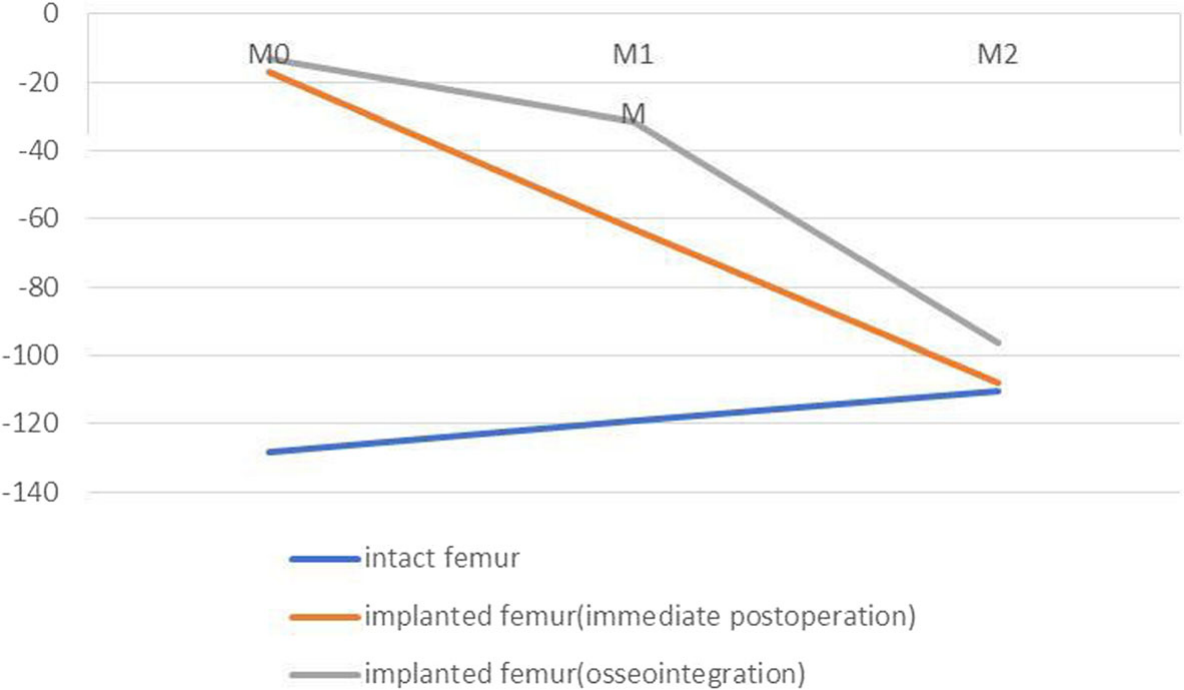
FE values of strain of intact and implanted femur at medial region

The differences in maximal principal strains between the implanted and intact femurs are presented in Figs. 5, 6, 7, and 8, which show varying degrees of stress shielding or stress concentrations in the anterior, posterior, lateral, and medial sides of the femur implanted with the sleeve and stem. Femoral arthroplasty with uncemented components led to marked alteration of the strain patterns under a simulated single-legged stance.

In the anterior aspect, maximal principal strains were reduced from distal to proximal in both immediate postoperative and osseointegrated implanted femurs (Fig. 5). Both immediate postoperative and osseointegrated implanted femurs produced marked reductions in the strains at the A0 femoral cortex compared with the intact state. The ITI ratios for the immediate postoperative and osseointegrated implanted femurs were 0.22 and 0.17, respectively. At A1, the ITI ratios for the immediate postoperative and osseointegrated implanted femurs were 0.55 and 0.35, respectively. At A2, the ITI ratios for the immediate postoperative and osseointegrated implanted femurs were 0.50 and 0.40, respectively. The ITI ratio differences at A0, A1, and A2 between immediate postoperative and osseointegrated implanted femurs were 0.05, 0.20, and 0.10, respectively. These findings showed that stress shielding was more severe in the osseointegrated implanted femur than in the immediate postoperative implanted femur and that the main strain change was located at A1 after osseointegration.

In the posterior aspect, the increase in maximal principal strains occurred from distal to proximal in both immediate postoperative and osseointegrated implanted femurs (Fig. 6). The ITI ratios were less than 1 for both immediate postoperative and osseointegrated implanted femurs at P0 and P1, and the ratios were 0.23 and 0.13 at P0 and 0.66 and 0.33 at P1, respectively. In contrast, the ITI ratios of both immediate postoperative and osseointegrated implanted femurs were greater than 1 at P2, with ratios 110% and 114%, respectively. The ITI ratio differences at P0, P1, and P2 between immediate postoperative and osseointegrated implanted femurs were 10%, 33%, and 4%, respectively. These findings also showed that stress shielding was more severe in the osseointegrated implanted femur than in the immediate postoperative femur and that the main strain changes were located at A1 after osseointegration.

In the lateral aspect, contrary to the trend of strain change in the intact femur, the increase in maximal principal strains occurred from distal to proximal in both immediate postoperative and osseointegrated implanted femurs (Fig. 7). The ITI ratios of the immediate postoperative and osseointegrated implanted femurs at L0 and L1 were was less than 1, with ratios of 28% and 21% at L0 and 74% and 44% at L1, respectively. In contrast, the ITI ratios of the immediate postoperative and osseointegrated implanted femurs were greater than 1 at L2, with ratios of 119% and 101%, respectively. The ITI ratio differences at L0, L1, and L2 between the immediate postoperative and osseointegrated implanted femurs were 7%, 30%, and 8%, respectively, These findings also showed that stress shielding was more severe in osseointegrated implanted femur than in the immediate-postoperative femur and that the main strain changes were located at L1 after osseointegration.

In the medial aspect, contrary to the trend of strain change in the intact femur, the reduction in maximal principal strains occurred from distal to proximal in both immediate postoperative and osseointegrated implanted femurs (Fig. 8). All ITI ratios of the immediate postoperative and osseointegrated implanted femurs were less than 1 at M0, M1, and M2, with ratios of 13%, 53%, and 98% for the immediate postoperative femur and 10%, 27%, and 87% for the osseointegrated femur, respectively. The ITI ratio differences at M0, M1, and M2 between the immediate postoperative and osseointegrated implanted femurs were 3%, 26%, and 11%, respectively. These findings also showed that stress shielding was more severe in the osseointegrated implanted femurs than in the immediate postoperative implanted femur and that the main strain changes were located at M1 after osseointegration.

### Maximum stresses in the components of the FE model

**Fig. 9:**
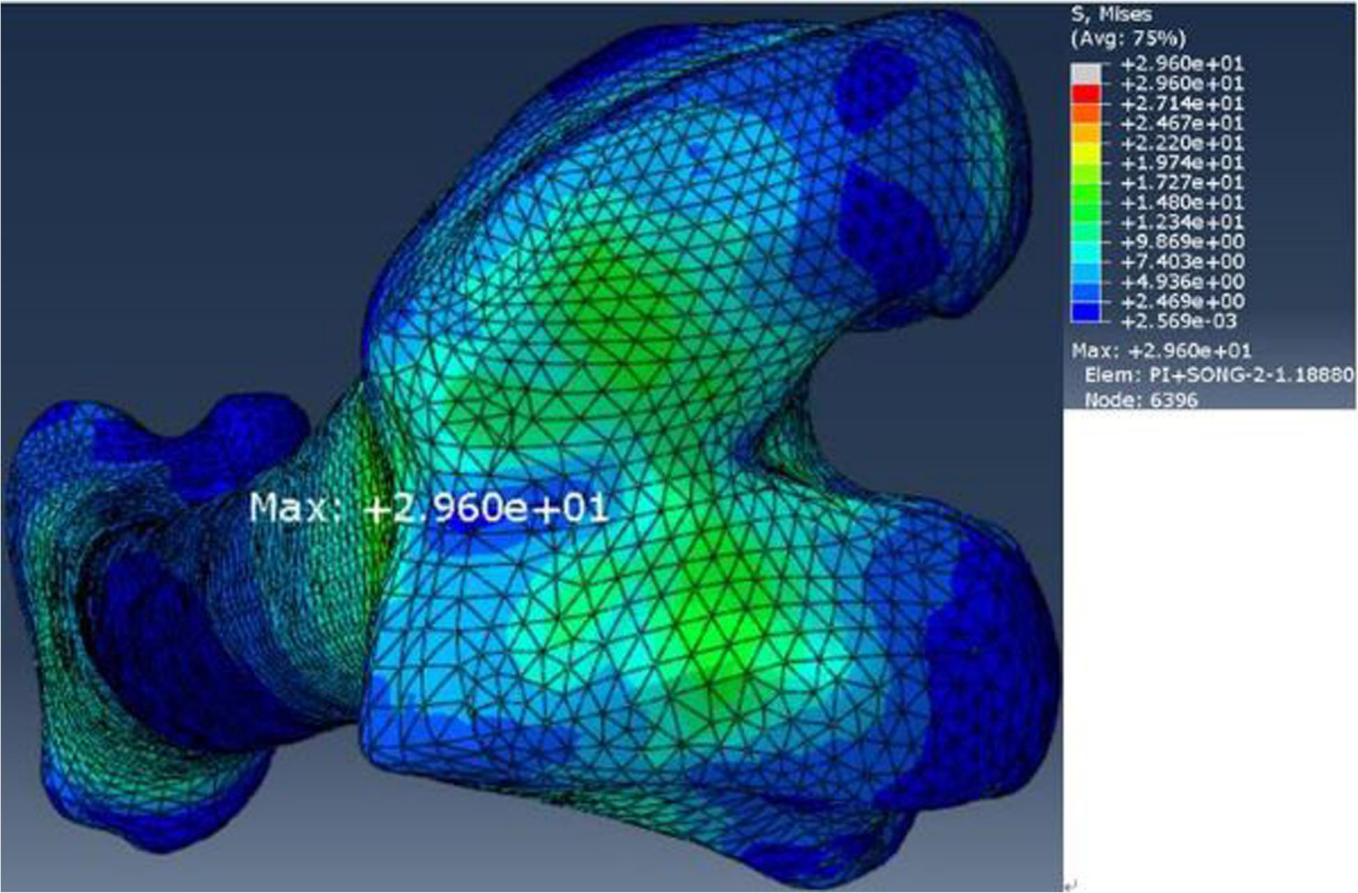
Maximal von Mises stress for the intact femur

**Fig. 10:**
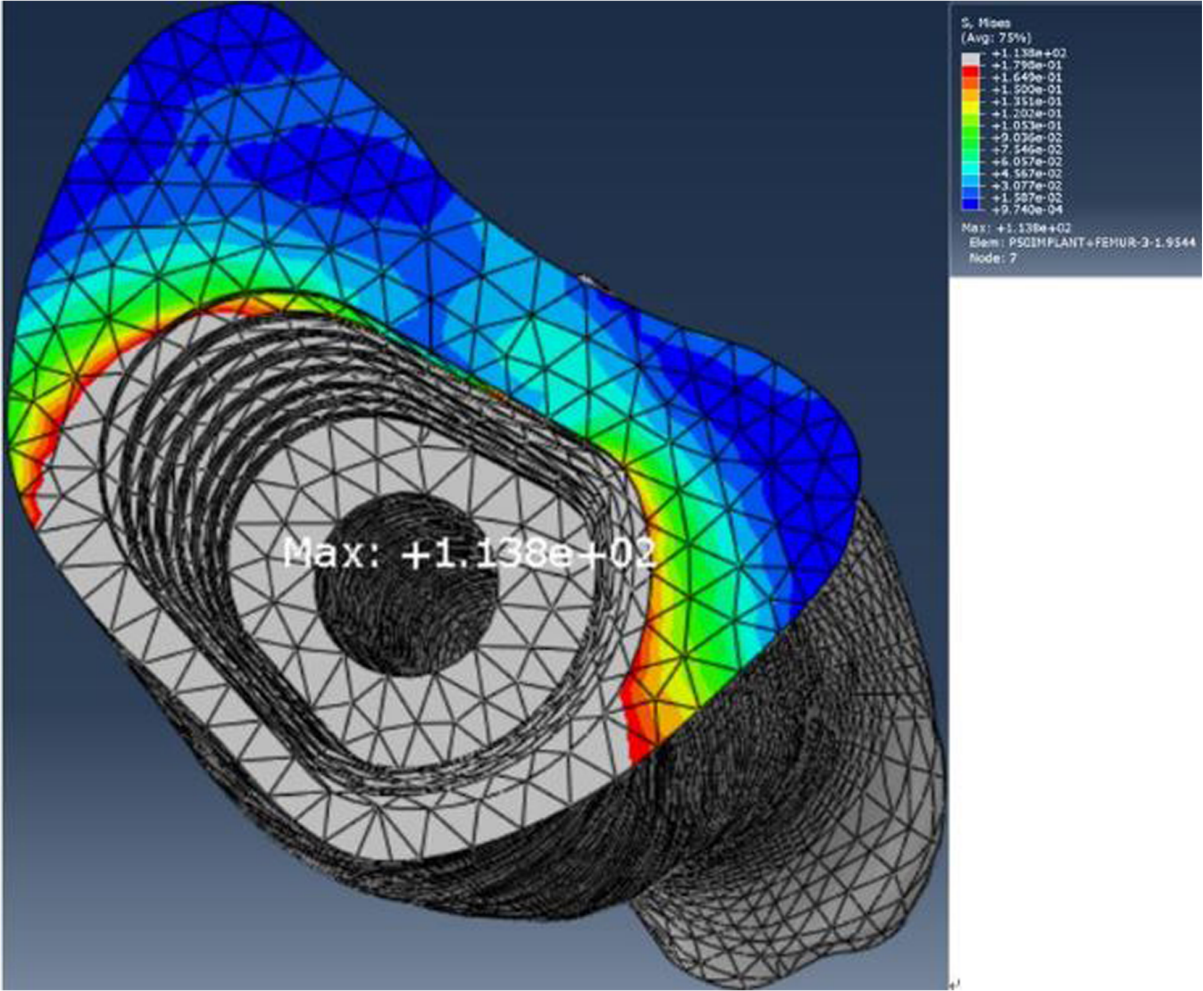
Maximal von Mises stress for the implanted femur in the immediate postoperative condition

**Fig. 11:**
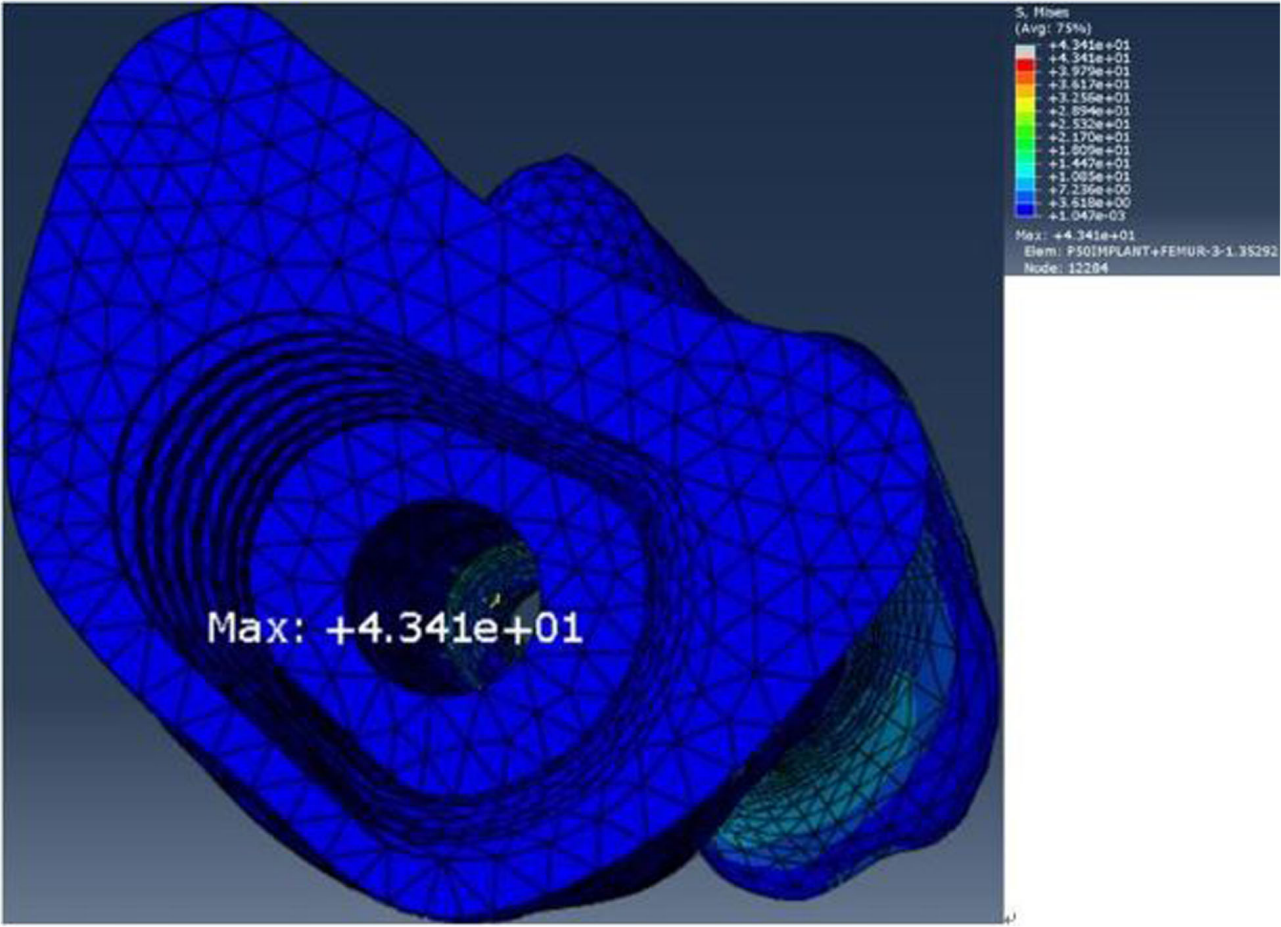
Maximal von Mises stress for the implanted femur in the osseointegrated condition

The maximum stresses of in the components of the FE model are listed in Table 6. The maximum stress for the FE model of the intact femur was located on the center of the femoral trochlear groove (Fig. 9). The maximum stress for the FE model of the implanted femur was located at the center of the posterior region of the endosteum at the level 2 plane which was approximately 175 mm from the knee joint line. The maximum stresses in the immediate postoperative implanted femur and osseointegrated femur was 3.8 times and 1.5 times larger than those in the intact femur, with stresses of 113.8 MPa and 43.41 MPa, respectively (Figs. 10 and 11). The maximum stresses in the sleeve were 27.29 MPa and 24.66 MPa in the immediate postoperative implanted femur and osseointegrated femur, respectively, which were located near in the anterolateral part of the sleeve-anchored cylinder interface. The maximum stresses in the stem were 221 MPa and 18.09 MPa in the immediate postoperative implanted femur and osseointegrated implanted femur, respectively, which were located at the end of the anterolateral flute near the tip of the stem.

**Table 6:** Maximal von Mises stresses of the femur, sleeve, and stem in single-legged stance (MPa)

	Intact femur	Implanted femur	Sleeve	Stem
Maximal stress	29.6	113.8^a^	27.29^a^	221^a^
		43.41^b^	24.66^b^	18.09^b^

^a^Data are obtained in the immediate postoperation condition

^b^Data are obtained in the osseointegration condition

## Discussion

The most important finding of the present study was that stress shielding still occurred in the distal femur implanted with a sleeve and stem, especially in the metaphysis, in both the immediate postoperative and the osseointegrated conditions. In addition, periprosthetic fracture in the single-legged stance condition could not occur in the immediate postoperative or osseointegrated implanted femurs.

Overall, a comparison of the numerical strains to the mean measured strains showed that the results from the FE model reasonably reflected the strain distribution on the femur with and without a megaprosthesis. The *R*^2^ value (*R*^2^ = 0.95 and 0.99, respectively) demonstrate this agreement, as these coefficients are close to the ideal value of 1. Furthermore, the RMSE values were approximately 10%, with 6% for the implanted femur and 14% for the intact femur. These results were within the range of those from previously published studies, similar to the *R*^2^ values. An excellent correlation between the FE analyses and experimental observations with an *R*^2^ value of 0.976 (RMSE% < 6%) was shown by Katz et al [35]. Completo et al. [29] reported that a linear regression between the numerical and mean experimental strains produced *R*^2^ values between 0.986 and 0.989 and RSME values between 8 and 14% in their study. In the previous work, Taddei et al. [36] reported a slightly lower fit between the FE-predicted strains and the experimental strains, with lower *R*^2^ value of 0.69 and 0.79, respectively, which partly explains why we concentrated the strain gauges in the metaphysis and diaphysis of the femur. However, particular attention was dedicated to the epiphyseal region in their work, where meshing with an automatic strategy is difficult.

Heterogeneity of the cortical and cancellous bones, which can the stress results, was not considered in our model, which is why the experimental results and simulation results showed some differences in the study. Fulvia et al. [22] and Yosibash et al [37] showed that an inhomogeneous model very accurately predicts the measured stress field in their studies. Furthermore, the presented model did not consider the known local anisotropic behavior of the bone tissue assigning an average Young’s modulus to each element. Fulvia et al. [22] and San et al [38] showed that introducing local anisotropy might further improve the predictive capabilities of the model. In addition, the RMSE values were lower for the implanted femur than for the intact femur, and we speculated that after implantation of the sleeve and stem, the degree of anisotropy decreases, tending towards more isotropic behavior, which is consistent with previous research [39].

The study showed that the femoral cortex was not impervious to the reconstruction technique and that sleeve and stem addition tended to reduce the magnitude of cortex strains. The maximum reduction for the femur implanted with a stem occurred at the distal level (level 0) relative to the intact femur. At level 0, stress shielding was most obvious in the medial region for both the immediate postoperative femur and the osseointegrated femur, followed by the anterior side of the immediate postoperative femur and the posterior region of the osseointegrated femur.

Stress shielding is relevant to many factors, including implant size and shape, material composition and bone size, and shape and density [40]. The elasticity of the implant was one of the key parameters that could influence femoral strain shielding. The elastic modulus ratio between the implant and bone was 110:16.7, as shown in this research. On the basis of beam theories described by Dujovne et al. [41], the stems were always stiffer axially than the corresponding femur, more metaphysially than diaphysially. Hence, obvious stiffness mismatches occurred distally, which may explain the prominently reduced strains obtained in the metaphysis of the femur (L0) after implantation with a noncemented sleeve and stem. In addition, the overall strain response was greatest in the medial aspect of the femur as a result of bending under single-legged stance loading [31].

At level 2, stress concentration was mainly found at P2 not only for the immediate postoperative femur but also for the osseointegrated femur. At P2, the stress concentration was strengthened by 4% from the immediate postoperation to osseointegrated conditions. In contrast, stress shielding was located at A2 for both the immediate postoperative femur and the osseointegrated femur. Bone loss due to distal stress transfer might be easier to locate in the anterior cortex of the femur, indicating eccentric osteopenia, which has been confirmed by some authors, including Chunlin et al. [7] and Rong-Sen [42].

The cortical bone strains at sliding interfaces were nearly higher than those at the bonded interfaces except for the location of P2. The main difference in the ITI ratios, which were between 20 and 33%, occurred at level 1. The characteristic strain change showed that the bonding conditions substantially influenced the predicted strains. Stress shielding in the distal part of the femur might be more severe in the osseointegrated condition than in the immediate postoperative condition. The mechanical effects of stem interface characteristics in arthroplasty were in agreement with those in previous studies. Van Lenthe et al. [43] and Huiskes [44] demonstrated that the cortical bone stresses around a press-fit stem were also higher than those around a bonded stem. McNamara et al. [45] studied the relationship between bone-prosthesis bonding and load transfer in total hip reconstruction and claimed that this greater stress shielding produced by the bonded stem was probably a result of greater initial stem-bone stability, which provided a more rigid system; hence, most of the physiological loading was transferred to the implant and away from the comparatively more compliant surrounding bone.

The maximal stress in the femur increased by 2.8 times from 29.6 to 113.8 MPa because of implantation of the sleeve and stem; these stresses are less than the strength of the femur, which is approximately 172–176 MPa [46]. The maximal stresses of the sleeve and stem were 27.29 and 221 MPa, respectively, which were far from 765 MPa for the yield strength of titanium alloy (Ti-6Al-4 V) at 0.2% offset reported by Todd et al. who also claimed that linear models demonstrate better predictive capabilities for Young’s modulus, yield strength, and especially ultimate tensile strength [47]. Consequently, implantation of the sleeve and stem in a single-legged stance theoretically could not induce femoral fracture or metal component breakage.

This study has several limitations. First, only a single femur was used where implants were simulated as being implanted in an ideal manner; thus, this study did not account for variability in bone shape or surgical imperfections. Therefore, caution should be exercised when interpreting the results presented herein. A future study with a larger sample size is required to analyze the effect of bone shape on the femoral strain distribution [48]. Second, the experiments and FE models are similar but not identical. The boundary and loading conditions in the FE model are precise, whereas variations inherently exist in any experimental setup [29], which that may change the load and stress distribution. Third, the FE analysis was carried out under a single loading configuration. Further investigation of the effects of other loading conditions might be necessary in the future [49]. Finally, the longer stem may lead to greater stress reduction in the cortical bone [11]; however, only one 10-cm stem was used in this study, which could strengthen the stress shielding induced by the sleeve.

## Conclusions

In conclusion, this study reveals that a metaphyseal sleeve may cause stress shielding relative to the intact femur, especially in the distal metaphysis. However, stress concentrations might occur at the lateral and posterior cortices around the tip of the stem, especially the posterior cortex during osseointegration. Although more stress shielding was identified in the osseointegrated conditions, the periprosthetic fracture risk may be lower after osseointegration than immediately postoperatively.

## Data Availability

Please contact the authors for data requests.

## Abbreviations

FE: Finite element
GCTB: Giant cell tumor of bone
ITI: Implanted to intact
*R*^2^: Coefficient of determination
RMSE: Root-mean-square error
M: Medial aspect of the femur
L: Lateral aspect of the femur
A: Anterior aspect of the femur
P: Posterior aspect of the femur
*S*_EXP_: Values of the experimental strain
*S*_FE_: Numerical principal strains
